# Adverse maternal and perinatal outcomes in women with previous preeclampsia: a prospective study

**DOI:** 10.1016/j.ajog.2011.02.014

**Published:** 2011-06

**Authors:** Kate Bramham, Annette L. Briley, Paul Seed, Lucilla Poston, Andrew H. Shennan, Lucy C. Chappell

**Affiliations:** Maternal and Fetal Research Unit, Division of Women's Health, King's College London School of Medicine, London, England, UK

**Keywords:** chronic hypertension, perinatal outcome, preeclampsia, recurrence, risk factor

## Abstract

**Objective:**

The purpose of this study was to assess recurrence rates of preeclampsia and neonatal outcomes in women with a history of preeclampsia that required preterm delivery.

**Study Design:**

Five hundred women with previous preeclampsia that required delivery at <37 weeks' gestation were followed prospectively.

**Results:**

Preeclampsia reoccurred in 117 women (23%). Predictive factors included black (odds ratio [OR], 2.29; 95% confidence interval [CI], 1.16–4.53) or Asian (OR, 2.98; 95% CI, 1.33–6.59) ethnicity, enrollment systolic blood pressure of >130 mm Hg (OR, 2.89; 95% CI, 1.52–5.50), current antihypertensive use (OR, 6.39; 95% CI, 2.38–17.16), and proteinuria of ≥2+ on enrollment urinalysis (OR, 12.35; 95% CI, 3.45–44.21). Women who previously delivered at <34 weeks' gestation were more likely to deliver preterm again (29% vs 17%; relative risk, 1.69; 95% CI, 1.19–2.40) than were those women with previous delivery between 34 and 37 weeks' gestation.

**Conclusion:**

Although this study confirms that women with previous preeclampsia that required early delivery are at high risk of the development of preeclampsia, the study identifies risk factors for recurrence and illustrates that women with previous preeclampsia are at greater risk of adverse neonatal outcome.

Preeclampsia is a multisystem disorder that complicates approximately 4-6% of pregnancies in the United Kingdom^1^ and is associated with maternal and fetal death and morbidity. Women with a history of preeclampsia have a higher risk of the development of preeclampsia in subsequent pregnancies,^2-7^ but the likelihood or recurrence is poorly defined; previous studies have either included a majority of women who delivered at >37 weeks' gestation in whom the risk of recurrence is low^8^ or have relied on information that was drawn from routinely collected clinical data in which the accuracy of diagnosis may be imprecise.^9^ Women with previous preeclampsia that required delivery at <34 weeks' gestation are of particular concern because it is recognized that they are at greater risk of recurrent disease and worse fetal outcome. The risk of the development of preeclampsia in these women is also uncertain because previous reports have been of atypical populations and/ or small numbers of women.^7,10^

Recurrent preeclampsia has also been associated with increased rates of preterm delivery, delivery of a small-for-gestational-age (SGA) infant, and perinatal death in women with recurrent preeclampsia when compared with preeclampsia in a first pregnancy.^3,10^ Although a higher rate of associated neonatal complications might be anticipated, this has not been investigated formally.

In this study, we report the results of a planned secondary analysis of women with a history of preeclampsia who were recruited as part of a prospective randomized control trial of vitamin C and E supplementation in women who were at increased risk of preeclampsia, for whom previous preeclampsia (delivery at <37 weeks' gestation) was 1 of 8 entry criteria.^11^ The objectives of this analysis were to determine the incidence of recurrent disease in women with previous preeclampsia and to identify predictive risk factors for subsequent preeclampsia. A further objective was to define the neonatal outcome in those who did and did not experience recurrent disease, in particular for those who previously delivered at <34 weeks' gestation.

## Materials and Methods

A randomized placebo controlled trial of vitamin C and E supplementation (the Vitamins in Preeclampsia trial, VIP no. ISRCTN 62368611) was completed between August 2003 and June 2005.^11^ All 2404 women who were recruited were considered to be at increased risk for the development of preeclampsia according to at least 1 of 8 defined criteria (Figure). They included 500 women with singleton pregnancies who had preeclampsia at <37 weeks' gestation in their most recent pregnancy.

After recruitment at 14^+0^-21^+6^ weeks' gestation, women were assigned randomly to receive placebo preparations or 1000 mg vitamin C and 400 IU vitamin E daily until delivery. There was no significant difference in the primary outcome of preeclampsia between women who received placebo or treatment. In subgroup analyses of women with previous preeclampsia, there were no significant differences in preeclampsia, low birthweight, or SGA infants in the control and intervention arms; consequently, data for women in the placebo and intervention arms were analyzed together. Trial participants with multiple pregnancies were excluded from the analysis.

Personal and demographic details, which were obtained at the enrollment visit, and pregnancy outcome were recorded in a customized secure password-protected internet-based study-specific database. Urinalysis was confirmed by a routine visual dipstick from a midstream urine sample that was collected by clinical midwives.

The South East Multi Ethics Research Committee provided ethics approval (no. 00/01/027), and site-specific approval was obtained from all participating centers.

### Definitions

Inclusion criteria for this analysis were previous *preeclampsia* that was defined as preeclampsia in the pregnancy preceding the index pregnancy that required delivery at <37 completed weeks' gestation. *Chronic hypertension* was defined as a diastolic blood pressure of ≥90 mm Hg (Korotkoff Stage 5) at the enrollment visit or at <20 weeks' gestation or the use of current or previous antihypertensive medication. For women with chronic hypertension and no proteinuria, a diagnosis of *preeclampsia* was defined as the new development of proteinuria in accordance with the International Society of Study of Hypertension in Pregnancy (ISSHP) guidelines.^12^ In women with preexisting proteinuria or hypertension, a diagnosis of preeclampsia was based on the development of gestational hypertension or proteinuria or after the identification of clinical or biochemical markers or at least 1 additional feature of preeclampsia (eg, HELLP [hemolysis, elevated liver enzymes, low platelets] syndrome or eclampsia). In women with both essential hypertension and preexisting proteinuria, the diagnosis was confirmed by 2 senior clinical staff members who acted independently who sought additional features of preeclampsia, as outlined in the ISSHP guidelines.^12^ *Severe preeclampsia* was defined according to ISSHP guidelines as diastolic blood pressure of >110 mm Hg with proteinuria as defined earlier.

Birthweights were assessed by customized birthweight percentile charts (www.gestation.net/birthweight_centiles/centile_online.htm); SGA was considered to be <10th percentile. The following available data were also used in analysis: preterm birth (<37 and <34 weeks' gestation, both spontaneous and iatrogenic), gestational age at delivery, perinatal death (intrauterine death at >24 weeks' gestation or postnatal by 7 days), antenatal inpatient nights and mode of delivery, and admission to neonatal unit.

### Statistical analysis

Categoric variables were summarized with the use of percentages and compared with the use of the χ^2^ test. Risk ratios with 95% confidence interval (CI) were calculated to determine the relationship between maternal and neonatal endpoints. Bootstrapping was used to develop confidence intervals for the difference in the arithmetic mean for indices of health care resources (maternal and neonatal inpatient stay). Risk factors for continuous outcome were analyzed by linear regression with robust standard errors and for binary outcome by logistic regression, which is expressed as odds ratio (OR) with 95% CI. A probability value of < .05 was determined to be statistically significant. Analysis was conducted with SPSS software (version 16; SPSS Inc, Chicago, IL) and Stata software (version 10.1; StataCorp, College Station, TX).

## Results

Baseline demographics and management characteristics are provided in Table 1 for the 500 women with previous preeclampsia, according to the development of recurrent preeclampsia that occurred in 117 women (23%). Table 2 gives a comparison of maternal and neonatal outcomes in women who had preeclampsia in the index pregnancy and those who did not. The one maternal death occurred in a woman with chronic hypertension and human immunodeficiency virus who had recurrent preeclampsia. The death was subsequent to and unrelated to the pregnancy.

Predictors of recurrent preeclampsia are shown in Table 3. Age, body mass index, smoking history, gestation of delivery in previous pregnancy for preeclampsia, chronic renal disease, antiphospholipid syndrome, and diabetes mellitus were not associated with increased risk of recurrent disease. Only women who required current hypertensive treatment were at greater risk for recurrent disease, whereas those women who previously required antihypertensive treatment were not (OR, 1.28; 95% CI, 0.77–2.14).

Of the women who experienced recurrent preeclampsia, those with previous delivery at <34 weeks' gestation (n = 76) were more likely to deliver an SGA infant (n = 54 [71%] vs = 12 [29%]; *P* < .0001) and to develop gestational hypertension earlier (n = 34.4 [interquartile range, 28.2–37.1] vs = 36.1 [interquartile range, 34.6–38.3]; *P* = .028). than were those women who delivered from 34-37 weeks' gestation (n = 41) to have preterm deliveries at <37 weeks' gestation (n = 54 [71%] vs = 16 [39%]; *P* = .001) and at <34 weeks' gestation (n = 31 [41%] vs = 5 [12%]; *P* = .001).

Similar risk factors were associated with preterm delivery (black ethnicity [OR, 3.03; 95% CI, 1.76–5.22], Asian ethnicity [OR, 4.25; 95% CI, 2.00–9.02]), systolic blood pressure of >140 mm Hg (OR, 2.41; 95% CI, 1.43–4.07), diastolic blood pressure of 80-90 mm Hg (OR, 1.96; 95% CI, 1.25–3.07), and diastolic blood pressure of >90 mm Hg (OR, 2.20; 95% CI, 1.13–4.30). Systolic blood pressure of >140 mm Hg (OR, 2.41; 95% CI, 1.43–4.07), diastolic blood pressure of 80-90 mm Hg (OR, 2.47; 95% CI, 1.65–3.71), diastolic blood pressure of >90 mm Hg (OR, 2.60; 95% CI, 1.39–4.87) were also risk factors for SGA infants.

Comparison of pregnancy outcome for women with a previous delivery for preeclampsia at <34 weeks' gestation and 34-37 weeks' gestation is shown in Table 4. For women with previous delivery at <34 weeks' gestation who did not experience preeclampsia, the risk of delivery of an SGA infant remained high (39/226; 17.3%). At recruitment, women who delivered at <34 weeks' gestation in a previous pregnancy were more likely to be taking prophylactic aspirin (187/304 [61.5%] vs 66/196 [33.7%]; *P* < .0001) or to be on antihypertensive therapy (55/304 [18.1%] vs 18/196 [9.2%]; *P* = .006) than were women who previously delivered at >34 weeks' gestation.

## Comment

In this prospective investigation, women with preeclampsia who delivered at <37 weeks' gestation in a previous pregnancy had a 1 in 4 risk (23%) of recurrent disease. Women with chronic hypertension comprised one-third of the women with previous preeclampsia who were recruited; this conferred significant additional risk. Adverse maternal and perinatal outcomes occurred not only in women who experienced preeclampsia again but also in those women who did not; preterm delivery and delivery of an SGA infant (by customized percentile) were higher than in the general population in both groups but were also more likely to occur in those women with recurrent disease than in those women who did not experience preeclampsia. Women with recurrent preeclampsia also had longer maternal inpatient stays; neonatal unit admission was also increased compared with women who did not have recurrent disease, which reflects the economic burden of the disease. Women who previously delivered at <34 weeks' gestation or from 34-37 weeks had similar rates of recurrence; women in the former group were more likely to have preterm deliveries and SGA infants.

Previous studies already have identified the low risk of recurrence in women with delivery at term in the previous affected pregnancy^8^; our trial focused on the clinically more important cohort of women with previous preterm delivery for whom risk of recurrence with associated adverse perinatal outcomes was likely to be high and who were relatively underrepresented in other studies.

Women with previous preeclampsia who had been recruited to 2 comparable studies of antioxidants for the prevention of preeclampsia, which included women with preeclampsia at <37 weeks' gestation, have reported similar risk (33%) or even higher risk (75% in developing countries) of recurrent disease.^13,14^ The recurrence rate in our study of 25% for women who previously had delivered at <34 weeks' gestation is also similar to that from a much smaller study of 120 women that included women with severe preterm preeclampsia.^7^

Previous studies, which include women with previous preeclampsia at any gestation, have either drawn on epidemiologic databases or are smaller cohort studies. Two recent studies in large population databases report a recurrence risk of 14% (19,960 women in Norway)^4^ and 15% (19,540 women in Sweden)^15^ but have limitations in the use of coded diagnoses of uncertain validity and unknown gestation of delivery in the previous pregnancy. Two cohort studies, in which a more accurate diagnosis of preeclampsia was made but without knowledge of the gestation of previous preeclampsia also report a lower recurrence risk (15% and 18%).^2,3,7,14^ Preeclampsia most commonly occurs at term; therefore, all 4 studies are likely to include a majority of women who experienced preeclampsia in the index pregnancy at >37 weeks' gestation, which is associated with a lower risk of recurrence^8^ and may have contributed to the low rates that were reported.

In one study of Australian women in which the definition of preeclampsia did not require proteinuria, Brown et al^6^ reported that women with nonproteinuric preeclampsia in their first pregnancy were more likely to experience nonproteinuric preeclampsia in their next pregnancy and that the recurrence rate was higher than among women with proteinuric preeclampsia in their first pregnancy. This highlights how variation in the definition of preeclampsia can also confound an accurate assessment of recurrence rates; many women in the studies referred to earlier would not have been diagnosed with preeclampsia because of the requirement of proteinuria. Moreover, cohorts have often been recruited from homogeneous populations, with underlying medical disorders excluded, with varying degree. Severity of preeclampsia (other than gestation of delivery) in an index pregnancy may also contribute to recurrence risk^8^; however, this usually relies on self-report and is difficult to define with accuracy.

Ethnicity may be an important determinant. Most previous reports that have addressed recurrence of preeclampsia have included almost exclusively white populations.^4-6^ In our study, black and Asian ethnicities were the only demographic predictor of recurrent preeclampsia. Black ethnicity has also been identified as a risk factor in women with early-onset recurrent preeclampsia in one other study.^10^ High rates of recurrent disease were reported in Brazilian women of mainly nonwhite ethnicity (37% recurrence)^13^ and in women from India, Peru, South Africa, and Vietnam (75% recurrence).^14^ This would suggest that ethnicity or factors that are related to ethnicity (such as indices of deprivation) contribute to a high rate of recurrence.

The population mix also represented pregnant women with previous preeclampsia in 25 centers across the United Kingdom in whom comorbidities are seen. These data are therefore generalizable to populations with a similar representation of other chronic diseases.

Chronic hypertension is an established risk factor for preeclampsia. Up to 50% of pregnancies in women with severe prepregnancy hypertension may be complicated by preeclampsia.^13,16,17^ We previously reported that 22% of women with chronic hypertension experienced preeclampsia.^6^ The difference in the recurrence rates between this and previous studies therefore may be determined in part by a high incidence of chronic hypertension. Only women who require antihypertensive treatment at booking, which may reflect more severe hypertension, were at increased risk of having recurrent preeclampsia. Other prospective studies that reported high rates of recurrent preeclampsia also included a high proportion of women with chronic hypertension at study entry (24% and 31%).^13^ Reports that describe lower rates of recurrent preeclampsia have low proportions of women with chronic hypertension,^5,18^ or the incidence of chronic hypertension is not reported.^4^

Women with chronic kidney disease have elevated rates of preeclampsia and superimposed preeclampsia, which increase with severity of renal impairment.^19^ Women with preeclampsia at <30 weeks' gestation are more likely to have underlying renal disease than those who experience preeclampsia later in pregnancy.^20^ In the present study, proteinuria (≥2+) at study entry was associated strongly with recurrent superimposed preeclampsia.

Preeclampsia and fetal growth restriction share underlying pathophysiologic abnormalities that include defective placentation because of inadequate trophoblast invasion, which results in reduced placental blood flow and associated endothelial dysfunction and angiogenic factor disequilibrium. Fetal growth restriction in a first pregnancy, without gestational hypertension, is also associated independently with hypertensive disorders in the next pregnancy.^21^ It is thus unsurprising that women with previous preeclampsia had a markedly increased risk of an SGA infant than background; a novel aspect of our study is the observation that delivery of an SGA infant occurred in 56% of those pregnancies with recurrent preeclampsia and 24% of subsequent pregnancies even without recurrence. This is likely to reflect shared pathophysiologic condition and the influence of other coexisting risk factors (eg, chronic hypertension) and may be the consequence of the inclusion of a high proportion of women who delivered at <34 weeks' gestation in their previous pregnancy and the use of customized percentiles that provide a more accurate detection of important adverse neonatal outcome.

Preterm delivery at <37 and <34 weeks' gestation was elevated in women with and without recurrent preeclampsia compared with the general population; however, rates were significantly higher in women with recurrent preeclampsia than in women without preeclampsia and in women who previously had delivered at <34 weeks' gestation than women who delivered later. The majority of preterm deliveries were iatrogenic. In general, women with preeclampsia that occurs at <37 weeks' gestation have worse perinatal outcomes than those who have preeclampsia at term, with higher rates of perinatal mortality, fetal growth restriction, and placental abruption.^22^ Several studies have identified previous preeclampsia as a significant risk factor for preterm delivery in future pregnancies and that these deliveries are predominantly because of medical intervention.^3,6,8,23-25^ Dukler et al also showed that severity of preeclampsia in primiparous women was correlated with preterm delivery in their subsequent pregnancies.^8^

This study has a number of strengths; it is one of the largest prospective analyses of women with preeclampsia in a previous pregnancy that has used independently confirmed diagnoses of recurrent disease. The individualized and well-defined diagnosis of preeclampsia without reliance on hospital coding statistics, which are known to be inaccurate, is an important strength. Inclusion of a high proportion of women with previous delivery at <34 weeks' gestation is of particular value to clinicians because previous data are sparse. This multicenter study from 25 units with a diverse spectrum with respect to age, ethnicity, body mass index, and additional risk factors enabled the results to be extrapolated to the wider population.

Additionally, this is one of the first studies to report pregnancy outcome in women with previous preeclampsia to have used customized birthweight percentiles to assess fetal growth restriction. This enables distinction to be drawn between an infant being constitutionally and pathologically SGA and provides a better surrogate measure for fetal growth restriction than standard growth indices.^26^

These data provide contemporaneous and accurate figures that should be of value in counseling women with previous preeclampsia that required delivery at <37 weeks' gestation and for guidance in management strategies. These strategies should focus on the identification of risk that is based on demographic and clinical factors at enrollment together with existence of comorbidities and target appropriate monitoring and prophylactic therapies while acknowledging that even those who do not develop recurrent preeclampsia may have a higher than background risk of adverse outcomes, such as SGA infants.

To date, no useful serologic predictors of preeclampsia in women with previous disease have been identified.^13^ We have highlighted potentially important clinical predictors of recurrent disease. Prophylactic strategies should focus on this group of women for secondary prevention.

Aspirin is currently the only recommended treatment to prevent the development of the condition; however, outcomes of those women with previous preeclampsia that was treated and not treated are varied.^7^ In this investigation, more women who had recurrent disease were taking aspirin for preeclampsia prevention than those who did not; however, the effect of aspirin cannot be determined because of the uncontrolled nature of prescribing (eg, women at higher risk may have been more likely to have received aspirin). Further work that will assess which subgroups of women would benefit from certain treatment (eg, aspirin) is necessary.

Long-term health risks in women with preeclampsia are now well-recognized and include increased incidence of hypertension, cardiovascular, cerebrovascular, and renal disease.^27-30^ Women with recurrent preeclampsia in their second pregnancies have been shown to have a significantly higher incidence of chronic hypertension during a 10-year follow-up period than did women with previous preeclampsia and normotensive second pregnancies.^31^ The implications of recurrent preeclampsia for future occurrence of other diseases that are known to be more prevalent in women with previous preeclampsia (eg, cardiovascular disease) are unknown and require further study.

## Figures and Tables

**FIGURE:**
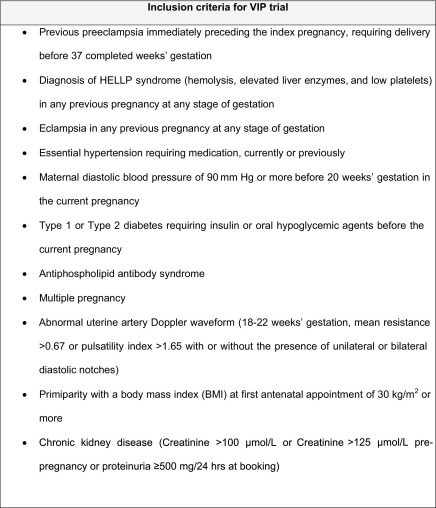
Inclusion criteria for vitamins in preeclampsia trial *Bramham. Obstetric outcomes with previous preeclampsia. Am J Obstet Gynecol 2011.*

**TABLE 1:** Baseline demographics according to outcome

Characteristic	No preeclampsia (n = 383; 77%)	Preeclampsia (n = 117; 23%)
Maternal age, y^a^	31.1 ± 5.5	31.9 ± 5.4
Gestational age at randomization, wk^b^	18.2 (15.7–20.6)	18.1 (15.6–20.4)
Ethnicity, n (%)		
White	309 (81)	72 (61)
Black	42 (11)	26 (22)^c^
Asian	23 (6)	14 (12)^d^
Other	9 (2)	5 (3)
Smoking status, n (%)		
Never	228 (60)	80 (68)
Current smoker (including occasional/social smoker)	46 (12)	10 (9)
Stopped before present pregnancy	82 (21)	21 (18)
Stopped during present pregnancy	27 (7)	6 (5)
Maternal weight, kg^a^	28.0 ± 6.1	27.9 ± 5.6
Body mass index, n (%)		
<25 kg/m^2^	137 (36)	41 (35)
25-30 kg/m^2^	122 (32)	43 (37)
31-35 kg/m^2^	71 (18)	19 (16)
≥35 kg/m^2^	53 (14)	14 (12)
Maternal baseline systolic blood pressure, mm Hg^a^	121 ± 15	127 ± 14^e^
<130 mm Hg, n (%)	265 (69)	58 (50)
130-139 mm Hg	64 (17)	31 (26)^f^
≥140 mm Hg	54 (14)	28 (24)^f^
Maternal baseline diastolic blood pressure, mm Hg	73 (10)	77 (10)^c^
<80 mm Hg, n (%)	253 (66)	55 (47)
80-89 mm Hg, n (%)	100 (26)	46 (39)^c^
≥90 mm Hg, n (%)	30 (8)	16 (14)^g^
Dipstick proteinuria		
Normal/trace	365 (95)	96 (82)
+	13 (3)	9 (8)^h^
≥ + +	5 (1)	12 (10)^e^
Additional risk factors at enrollment, n (%)^i^		
Previous HELLP (hemolysis, elevated liver enzymes, low platelets) syndrome	40 (10)	6 (5)
Previous eclampsia	28 (7)	5 (4)
Diabetes mellitus	15 (4)	3 (3)
Chronic kidney disease	3 (0.8)	4 (3.4)
Antiphospholipid syndrome	6 (1.6)	1 (0.9)
Chronic hypertension	112 (29)	49 (42)
Medication at enrollment, n (%)		
Aspirin	182 (48)	71 (61)^d^
Heparin	6 (1.6)	0
Current antihypertensive use	41 (11)	32 (27)^e^
Previous antihypertensive use	67 (18)	25 (21)
Subsequent additional medication after enrollment, n (%)		
Oral antihypertensive use	46 (58)	53 (68)
Parenteral antihypertensive use	1 (0.3)	8 (6.8)^e^
Magnesium sulfate use	5 (1.3)	20 (17.1)^e^

Bramham. Obstetric outcomes with previous preeclampsia. Am J Obstet Gynecol 2011.

a Data are given as mean ± SD;

b Data are given as median (interquartile range);

c *P* = .001;

d *P* = .008;

e *P* < .001;

f *P* = .002;

g *P* = .009;

h *P* = .03;

i Women with >1 risk factor were included in each relevant risk category.

**TABLE 2:** Maternal and neonatal outcome of women with recurrent preeclampsia vs women without preeclampsia in current pregnancy

Clinical feature	Preeclampsia (n = 117; 23%)	No preeclampsia (n = 383; 77%)	Risk ratio (95% CI)
Maternal outcomes			
Eclampsia, n (%)	3 (3)	0	
HELLP (hemolysis, elevated liver enzymes, low platelets) syndrome, n (%)	2 (2)	0	
Antepartum hemorrhage, n (%)	3 (2)	1 (0.3)	9.82 (1.03–93.52)
Spontaneous vaginal delivery, n (%)	24 (21)	140 (37)	0.56 (0.38–0.82)
Instrumental delivery, n (%)	5 (4)	33 (9)	0.50 (0.20–1.24)
Cesarean delivery, n (%)	88 (75)	210 (55)	1.37 (1.19–1.58)
Elective	78 (67)	156 (41)	1.64 (1.37–1.95)
Emergency	10 (8)	54 (14)	0.61 (0.32–1.15)
Total inpatient stay, d^a^	12.97 ± 9.29	5.06 ± 6.56	5.91 (4.36–7.48)^b^
Antenatal	7.99 ± 8.05	2.07 ± 5.85	7.91 (6.09–9.72)^b^
Postnatal	4.97 ± 3.84	2.99 ± 2.27	1.99 (1.24–2.73)^b^
Neonatal outcomes			
Preterm birth: <37 weeks' gestation, n (%)	70 (57)	53 (43)	4.32 (3.23–5.78)
Spontaneous	3 (3)	14 (4)	0.75 (0.26–2.11)
Iatrogenic	67 (54)	39 (39)	5.62 (4.02–7.87)
Preterm birth: <34 weeks' gestation, n (%)	36 (31)	22 (6)	3.39 (2.56–4.49)
Perinatal death, n (%)	6 (6)	12 (3)	1.63 (0.63–4.27)
Admission to neonatal unit or special care baby unit, n (%)	49 (42)	40 (10)	4.01 (2.79–5.76)
Inpatient stay in neonatal unit or special care baby unit, d^a^	14.7 ± 27.18	1.35 ± 6.01	5.96 (1.55–6.37)^b^
Intraventricular hemorrhage, n (%)	4 (3)	1 (0.2)	13.09 (1.48–116.0)
Birthweight			
<5th birthweight percentile, n (%)	63 (54)	56 (15)	3.68 (2.74–4.94)
Birthweight, kg^c^	2.32 (1.54–3.01)	3.22 (2.86–3.63)^d^	
Birthweight percentile^c^	2.5 (0–47.5)	36.4 (10.4–72.4)^d^	

*CI*, confidence interval.

Bramham. Obstetric outcomes with previous preeclampsia. Am J Obstet Gynecol 2011.

a Data are given as mean ± SD;

b Mean difference; confidence intervals calculated by bias-corrected random bootstrap, with 10,000 replications;

c Data are given as median (interquartile range);

d *P* < .001.

**TABLE 3:** Effect of baseline characteristics on the recurrence of preeclampsia

Baseline characteristics	Odds ratio	
Unadjusted^a^ (95% CI)	Adjusted^b^ (95% CI)
Ethnicity		
White	1.00 (Reference)	
Black	2.66 (1.53–4.62)	2.29 (1.16–4.53)^a^
Asian	2.61 (1.28–5.33)	2.98 (1.33–6.59)^b^
Other	2.38 (0.78–7.33)	3.20 (0.97–10.62)
Systolic blood pressure, mm Hg		
<130	1.00 (Reference)	
130-139	2.21 (1.32–3.70)	2.89 (1.52–5.50)^c^
≥140	2.37 (1.38–4.06)	2.38 (1.17–4.83)^d^
Diastolic blood pressure, mm Hg		
<80	1.00 (Reference)	
80-89	2.12 (1.34–3.33)	1.13 (0.62–2.06)
≥90	2.45 (1.25–4.81)	0.98 (0.35–2.75)
Current antihypertensive use	3.14 (1.87–5.28)	6.39 (2.38–17.16)^e^
Dipstick proteinuria		
Normal/trace	1.00 (Reference)	
+	2.63 (1.09–6.34)	1.73 (0.61–4.91)
≥++	9.12 (3.14–26.53)	12.35 (3.45–44.21)^e^

Estimates were adjusted for age, maternal body mass index, ethnicity, smoking, and maternal booking diastolic and systolic blood pressure at booking, previous and current antihypertensive therapy, and additional risk factors for trial entry that included antiphospholipid syndrome, chronic kidney disease, and diabetes mellitus.

*CI*, confidence interval.

Bramham. Obstetric outcomes with previous preeclampsia. Am J Obstet Gynecol 2011.

a *P* = .016;

b *P* = .008;

c *P* = .001;

d *P* = .017;

e *P* < .001.

**TABLE 4:** Maternal and neonatal outcome of women with early delivery for preeclampsia (<34 weeks' gestation in last pregnancy) vs women with delivery for preeclampsia from 34-37 weeks' gestation in last pregnancy

Clinical features	Previous delivery for preeclampsia		Risk ratio (95% CI)
<34 wk (n = 304)	34-37 wk (n = 196)
Maternal outcome			
Hypertensive disorders, n (%)			
Recurrent preeclampsia	76 (25)	41 (21)	1.05 (0.96–1.16)
Severe preeclampsia	30 (10)	6 (3)	1.08 (1.03–1.12)
Gestational hypertension	123 (40)	75 (38)	1.04 (0.90–1.20)
Severe gestational hypertension	39 (13)	10 (5)	1.09 (1.03–1.15)
HELLP (hemolysis, elevated liver enzymes, low platelets) syndrome, n (%)	1 (0.3)	1 (0.5)	1.00 (0.99–1.01)
Eclampsia, n (%)	1 (0.3)	2 (1)	0.99 (0.98–1.01)
Mode of delivery, n (%)			
Spontaneous vaginal	78 (26)	86 (44)	0.75 (0.66–0.87)
Cesarean section	202 (66)	98 (50)	1.49 (1.21–1.84)
Elective	161 (53)	73 (37)	1.33 (1.14–1.57)
Emergency	41 (13)	23 (12)	1.02 (0.95–1.09)
Other outcomes, d^a^			
Total inpatient stay	7.5 ± 7.9	6.0 ± 8.1	1.49 (–0.08 to 2.26)^b^
Antenatal	3.8 ± 6.7	2.9 ± 7.1	0.88 (–0.48 to 2.04)^b^
Postnatal	3.7 ± 2.9	3.1 ± 2.8	0.61 (0.10–1.10)^b^
Neonatal outcome			
Preterm birth: <37 weeks, n (%)	89 (29)	34 (17)	1.69 (1.19–2.40)
Iatrogenic	77 (25)	29 (15)	1.14 (1.05–1.25)
Spontaneous	12 (4)	5 (3)	1.04 (0.99–1.08)
Preterm birth: <34 weeks, n (%)	47 (15)	11 (6)	1.11 (1.05–1.18)
<5th birthweight percentile, n (%)	91 (30)	28 (14)	1.22 (1.11–1.34)
Admission to neonatal unit/special care baby unit, n (%)	70 (23)	19 (10)	1.46 (1.31–1.62)
Inpatient stay in special care baby unit, d^a^	6.0 ± 17.8	9.6 ± 2.1	3.96 (1.57–6.40)^b^

Bramham. Obstetric outcomes with previous preeclampsia. Am J Obstet Gynecol 2011.

a Data are given as mean ± SD;

b Mean difference; confidence intervals were calculated by bias-corrected random bootstrap, with 10,000 replications.
